# Opposite changes in the expression of clathrin and caveolin-1 in normal and cancerous human prostate tissue: putative clathrin-mediated recycling of EGFR

**DOI:** 10.1007/s00418-023-02183-8

**Published:** 2023-03-04

**Authors:** Boyu Xie, Hawra Zuhair, Rui Henrique, Michael Millar, Timothy Robson, Christopher Thrasivoulou, Kerry Dickens, Jane Pendjiky, Asif Muneer, Hiten Patel, Aamir Ahmed

**Affiliations:** 1 Centre for Stem Cells and Regenerative Medicine, King’s College London, London, UK; 2 Department of Pathology and Cancer Biology and Epigenetics Group, Portuguese Oncology Institute of Porto and Department of Pathology and Molecular Immunology, Abel Salazar Institute of Biomedical Sciences, University of Porto, Porto, Portugal; 3 Queen’s Medical Research Institute, University of Edinburgh, Edinburgh, UK; 4 Research Department of Cell and Developmental Biology, The Centre for Cell and Molecular Dynamics, Rockefeller Building, University College London, London, UK; 5 Division of Surgery, University College Hospital, London, UK; 6 Department of Urology, University Hospital of North Norway, 9038 Tromsö, Norway

**Keywords:** Prostate, Cancer, Clathrin, Caveolin, EGFR, Endocytosis

## Abstract

Endocytosis, an important macromolecule uptake process in cells, is known to be dysregulated in cancer. Clathrin and caveolin-1 proteins play a major role in receptor-mediated endocytosis. We have used a quantitative, unbiased and semi-automated method to measure in situ protein expression of clathrin and caveolin-1 in cancerous and paired normal (cancer adjacent, non-cancerous) human prostate tissue. There was a significant (*p* < 0.0001) increase in the expression of clathrin in prostate cancer samples (*N* = 29, *n* = 91) compared to normal tissue (*N* = 29, *n* = 67) (*N* = number of patients, *n* = number of cores in tissue arrays). Conversely, there was a significant (*p* < 0.0001) decrease in expression of caveolin-1 in prostate cancer tissue compared to normal prostate tissue. The opposite change in expression of the two proteins was highly correlated to increasing cancer aggressiveness. There was also a concurrent increase in the expression of epidermal growth factor receptor (EGFR), a key receptor in carcinogenesis, with clathrin in prostate cancer tissue, indicating recycling of EGFR through clathrin-mediated endocytosis (CME). These results indicate that in prostate cancer, caveolin-1-mediated endocytosis (CavME) may be acting as a brake and increase in CME may facilitate tumorigenicity and aggressiveness of prostate cancer through recycling of EGFR. Changes in the expression of these proteins can also potentially be used as a biomarker for prostate cancer to aid in diagnosis and prognosis and clinical decision-making.

**Supplementary Information:**

The online version contains supplementary material available at 10.1007/s00418-023-02183-8.

## Introduction

Receptor-mediated endocytosis (RME) allows internalization of specific macromolecules by a cell from the extracellular space by first binding to a specific cell receptor found in clathrin-coated pits of the cell membrane [reviewed in (Khan and Steeg 2021)]. Clathrin, so named to indicate a lattice-like structure (Pearse 1976), forms a triskelion shape made up of three heavy and three light chains with two isoforms identified for each chain (Brodsky 1985; Kaksonen and Roux 2018). The clathrin pits bud to form clathrin-coated vesicles with the bound receptor ligand complex, and the process is termed clathrin-dependent or clathrin-mediated endocytosis (CME). CME has been known to play a key role in cancer and clathrin is overexpressed in lung, liver and ovarian cancer (Seimiya et al. 2008; Smith et al. 2013; Wei et al. 2019).

In addition to CME there are other, clathrin-independent endocytotic processes, such as caveolae-dependent endocytosis (CavME), which is driven by integral membrane proteins called caveolins (Rothberg et al. 1992). Caveolae are small (50–100 nm) plasma membrane invaginations containing oligomeric integral membrane proteins called caveolins and peripheral membrane proteins collectively termed cavins (Hansen and Nichols 2010; Yamada 1955). Three isoforms of caveolins, ranging in molecular weight from 18 to 22 kDa, encoded by *CAV1*, *2* and *3* genes, are known. In addition to their role in clathrin-independent endocytosis, caveolins are also involved in intracellular Ca^2+^ signaling and mechano-transduction (Del Pozo et al. 2021; Gilbert et al. 2016). Both CME and CavME have been shown to play an important role in carcinogenesis and cancer metastasis. It has been suggested that the deregulation of endocytosis is likely to be a hallmark in many cancers (Mellman and Yarden 2013).

Prostate cancer is the second leading cancer in men worldwide (Ferlay et al. 2021); it is curable when confined to the organ but is a major cause of mortality when metastasized. It is known that dysregulation of receptor-mediated cell signaling pathways may play a role in both carcinogenesis and metastasis of prostate cancer (Baetke et al. 2012; Murillo-Garzon and Kypta 2017). There is, however, patchy information regarding the expression of clathrin or caveolin-1 in human prostate tissue. There are some studies of caveolin expression in human prostate tissue showing association of caveolin-1 with cancer progression and recurrence (Karam et al. 2007; Satoh et al. 2003; Yang et al. 2005, 1998). There is also some evidence from investigations of mechanisms in cell lines. For example, Prescott and Tindall (1998) observed androgen-induced clathrin mRNA and protein overexpression in LnCaP prostate cancer cell line (Prescott and Tindall 1998). It is thought that at a cellular level, clathrin also promotes prostate cancer cell proliferation and migration by binding to cadherin-11 and prostate-specific membrane antigen (Goodman et al. 2007; Satcher et al. 2015).

Extracellular ligand and membrane-bound receptor-dependent cell signaling pathways such as EGFR, Wnt, Notch, Hedgehog and NF-κB are critical during the process of carcinogenesis and metastasis (Nwabo Kamdje et al. 2017; Yang et al. 2020). Endocytosis is a major regulator for receptor availability for these signaling pathways. Receptor availability on the cell membrane can enhance or reduce the activity of key proliferative pathways (Khan and Steeg 2021; Mellman and Yarden 2013). If RME instead of degradation recycles the receptor, this may result in increased cell proliferation or carcinogenesis. There is evidence that EGFR is subject to such a regulation via CME (Chi et al. 2011). However, little information regarding the role of endocytosis-mediated receptor regulation for carcinogenic pathways such as EGFR exist, particularly in human tissue and in prostate cancer.

Furthermore, CME and CavME, like many other cellular processes, function in parallel, and it is therefore important to investigate the relationship between the expression of clathrin and caveolin-1, simultaneously, in human prostate cancer tissue. To investigate the molecular basis of CME and CavME in prostate cancer, we have used a multi-label immunohistochemical technique, combined with unbiased quantitative analysis (Arthurs et al. 2017; Symes et al. 2013), to simultaneously measure the expression of clathrin and caveolin-1, in normal and paired cancerous prostate tissue with different Gleason grades on tissue arrays (TAs). We tested the hypothesis that RME is increased in prostate cancer and is correlated to cancer progression. We also hypothesized that there is increased recycling of EGFR in prostate cancer which may be correlated to CME. Our results show a concurrent and opposite change in the expression of these clathrin and caveolin-1 proteins in prostate cancer and as the cancer progresses towards aggressive disease. We further show that both clathrin and EGFR colocalize with the adaptor protein 2 (AP2), a marker for endocytotic clathrin complexes (Gaidarov et al. 1996), and there is no change in the interaction between caveolin-1 and EFGR but there is a significant increase in EGFR colocalization with clathrin in prostate cancer tissue compared to normal. These results provide novel information regarding the expression and putative concurrent function of CME and CavME in prostate cancer development and progression. An important consequence of increased CME may be increased carcinogenesis via receptor recycling for key signaling pathways such as EGFR.

## Materials and methods

### Ethical approval and prostate tissue samples

Ethical approval (REC Ref 13/ES/0092) was granted by the joint research office University College London and University College Hospital through East of Scotland Research Ethics Service. All archival prostate tissue samples were collected via prostatectomy and used primarily for pathological analysis according to international guidelines in Portuguese Oncology Institute of Porto (Porto, Portugal) and were subsequently used to make tissue arrays utilized in this study. Samples were collected from patients with a median age of 60 (interquartile range 54, 65) years. Serum PSA levels (ng/ml) at diagnosis were also recorded as 8.32 (interquartile range 5.80, 10.43). The details of anonymized clinical information are described in Supplementary Table 1. A total of 29 individual prostate tissue samples classified by an expert uropathologist as cancerous and further sub-classified for Gleason scores were used; 29 cancer adjacent, non-cancerous tissue samples were also extracted. The experiment was conducted as a double-blind study with neither the experimenter (who performed antibody staining) nor the data analysts being aware of identity of the condition of the tissue samples or that of the antibody used. The data were only decoded after the anonymized quantitative analysis was completed (Symes et al. 2013).

### TA construction

TAs were constructed in our laboratory using a manual tissue arrayer (MTA1, Beechers Instruments, Sun Prairie, WI, USA) as described in detail elsewhere (Nariculam et al. 2009; Symes et al. 2013). Individual tissue cores (1 mm diameter) were extracted from the marked regions of prostate cancer and cancer adjacent (normal) prostate tissue blocks and moved to a recipient block using a Beechers MTA1 manual tissue arrayer (Beechers Instruments, Sun Prairie, WI, USA). Five recipient blocks were populated with samples (five to six tissue samples) from both prostate cancer (*N* = 29, *n* = 91) and cancer adjacent region (*N* = 29; *n* = 67) of the whole tissue blocks (*N* = number of patients, *n* = number of cores). Each individual tissue core was also examined by uropathologist to assess the Gleason grades (Supplementary Table 1). The following distribution of Gleason grades were used: 3 + 3 (*n* = 15); 3 + 4 (*n* = 4); 4 + 3 (*n* = 11); 4 + 4 (*n* = 43); 4 + 5/5 + 4 (*n* = 22).

TA blocks were sectioned at 6–8 μm thickness on glass slides using a Thermo Scientific™ HM355S automatic microtome. Sections on slides were stained with hematoxylin and eosin (H&E) and imaged at 40× magnification using NanoZoomer Digital Pathology Scanner 2.0 RS (Hamamatsu, Hertfordshire, UK) and re-examined by the pathologist.

### Immunohistochemistry

Multiplex staining of TAs, using anti-clathrin (heavy chain) (Abcam, cat. no. ab21679), anti-caveolin-1 (Abcam, cat. no. ab2910) and EGFR (Leica, NCL-EGFR) antibodies. Furthermore, anti-clathrin, EGFR and anti-AP2 (Sigma, A7107) antibodies on different TA sections were also tested. Immunohistochemical experiments were conducted using Bond Max automated staining system (Leica, UK) (Arthurs et al. 2020; Symes et al. 2013). Each antibody was optimized for pH and concentration dependence, antigen retrieval and temperature parameters; nuclei were counterstained with 4′,6-diamidino-2-phenylindole (DAPI). Antibodies were used at a dilution of 1:2000, 1:4000, 1:20 and 1: 500 for anti-clathrin, anti-caveolin-1, EGFR and anti-AP2, respectively. For multilabel experiments with clathrin, caveolin-1 and EGFR, TAs were labeled with Opal-650 (clathrin), Opal-520 (caveolin-1) and Opal-570 (EGFR), respectively; for multilabel experiments with clathrin, EGFR and AP2, the antibodies were labeled with Opal-650 (clathrin), Opal-520 (AP2) and Opal-570 (EGFR), respectively.

### Imaging of immunofluorescently labeled TAs

Tissue cores from multi-labeled immunofluorescence TAs were imaged using AxioScan Z1 scanner (Zeiss, Cambridge, UK) at 20× magnification for protein expression analysis, as described previously (Arthurs et al. 2017; Arya et al. 2015; Symes et al. 2013). The excitation/emission settings were 353/465 nm (DAPI); 493/517 nm (Opal-520, caveolin-1 or AP2), 653/668 nm (Opal-650, clathrin) and 550/570 nm (Opal-270, EGFR). Fluorescent signals were optimized to avoid saturation and for comparative analysis. The power settings and integration times of Hamamatsu ORCA Flash4 camera (Hamamatsu Photonics, Japan) on AxioScan were kept constant for all tissue cores imaged. For standardization, all imaging was conducted on the same day with all other image settings kept the same. Images of individual tissue cores (scenes in AxioScan Z1) were extracted from multiple 5712 × 5712 pixel, 300 dpi, 24-bit single color images and stored as tiff files.

For the measurement of colocalization coefficients (Global Intersection Coefficient, GIC) between different fluorescent labels, high magnification confocal imaging on a Leica SP8 microscope (Leica, UK) with a 63 × 1.4-NA oil objective and 6× optical zoom at a 1024 × 1024 pixel format was also conducted on sub-sets of different prostate tissue samples. The Z-stack scan was performed at 0.17 μm (at 600 Hz), yielding approximately 26–30 Z-sections for each tissue core largely including epithelial areas of prostate tissue.

### Quantitation of clathrin and caveolin-1 signal in prostate tissue

The expression levels for clathrin and caveolin-1 were quantified using an unbiased quantitative analysis developed in our laboratory (Arya et al. 2015; Symes et al. 2013) using an adapted ImageJ plugin (Rasband 2011). Briefly, the images were separated into red (Opal 650, clathrin), green (Opal 520, caveolin-1) and yellow (Opal-570, EGFR) channels and were exported from ZEN 3.1 (blue edition) software, opened in ImageJ software and converted to 8-bit grayscale; a threshold and segmentation routine was then applied. Lower and upper threshold parameters were set to include the median to maximum gray values of a range of samples across the sample cohort. The gray values were then used to perform semi-automated analysis of the whole tissue array. A similar approach was implemented to quantify the amount of tissue in each core, and this value was divided by the intensity to obtain a standardized signal for each label in prostate tissue samples.

Statistical analysis was performed using MedCalc software (version 19.7.2, www.medcalc.org) using Mann-Whitney *U* test, and boxplots were made using both GraphPad Prism 9 software (GraphPad Software, CA, USA) and Origin pro software (OriginLab, MA, USA).

### Deconvolution and colocalization analysis using high magnification confocal images

One hundred fifty-eight individual tissue cores (67 normal, 91 malignant cores including 15 Gleason score 3 + 3, 4 Gleason score 3 + 4, 7 Gleason score 4 + 3, 43 Gleason score 4 + 4 and 22 Gleason score 4 + 5 with at least 4 ROIs for each core) were identified for areas of cancer visually on the serial H&E section of the tissue array. These areas were approximated on the fluorescently labeled tissue array, and tissue cores were imaged using a Leica SP8 confocal system as described above.

The resulting image files were deconvolved using Huygens Professional software (version 20.04, Netherlands). Deconvolved images were saved as Huygens-specific (HDF5) files for each channel [excitation/emission (nm) DAPI = 405/429, Opal-520 = 488/517, Opal-570 = 561/595 and Opal-650 = 635/668]. The HDF5 files were imported into Huygens co-localization module and were background subtracted using ‘Gaussian minimum’ method; up to 4–16 regions of interest/image were analyzed to calculate the global intersection coefficient for co-localization (Arthurs et al. 2020). Colocalization analysis was conducted using Huygens professional software to calculate GICs.

## Results

### Histopathological analysis

An expert uropathologist diagnosed and marked the tissue sections (Supplementary Fig. 1) from which normal (cancer adjacent, non-cancerous) and cancerous prostate tissue were extracted for making tissue arrays. Subsequent to tissue array construction, the cores were verified and validated for diagnosis, including grading in each core, using H&E sections (Supplementary Fig. 2).

### Comparison of expression of clathrin and caveolin-1 in normal and cancer prostate tissue

**Fig. 1:**
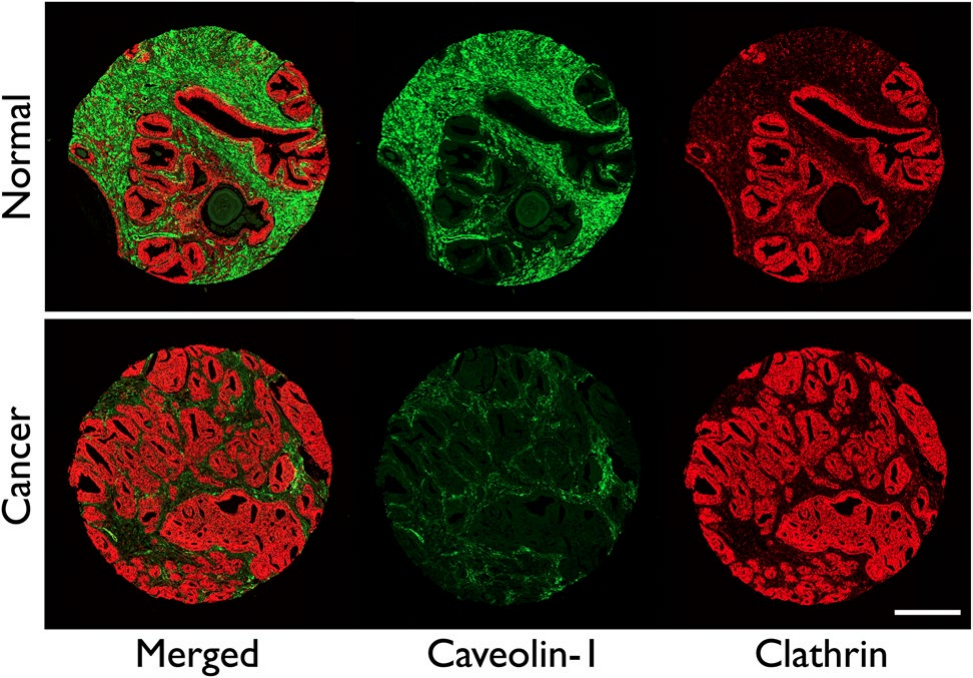
Representative images of normal (cancer adjacent, non-cancerous, top panel) and cancerous prostate tissue core (bottom panel) showing expression of caveolin-1 (green) and clathrin (red) proteins. For both normal and cancerous samples, composite images and individual channels Opal-520 (green) and Opal-650 (red) are also shown; individual channel images were used for quantitative analysis of expression of two proteins. Tissue arrays were imaged at 20 × magnification using an AxioScan Z1 (Zeiss). All settings were kept the same for subsequent comparative analysis. Scale bar: 200 μm

**Fig. 2:**
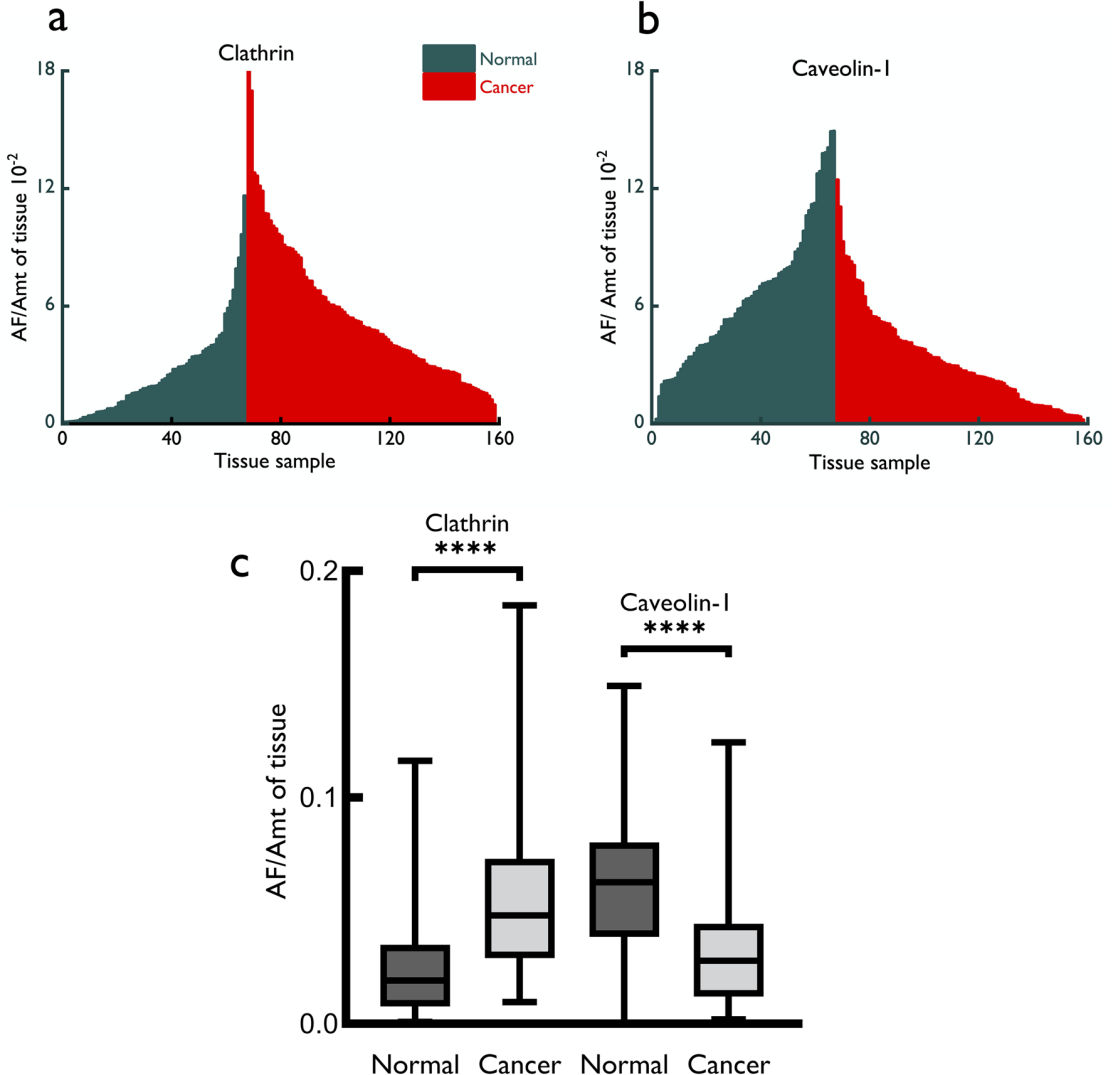
Mountain plots of population analysis for the expression of clathrin (**a**) and caveolin-1 (**b**) from the data obtained from ImageJ based quantitative analysis. Each line represents the value of individual tissue samples (normal, green; cancer, red). Results are given for the intensity signal expressed as area fraction (AF) divided by the amount (Amt) of tissue (in each tissue core) and fitted to a probit regression. Area fraction (AF) was divided by the total amount (Amt) of tissue in a core to standardize the data for the amount of tissue present in each core on the arrays. The data are also expressed to show quantitative expression of clathrin and caveolin-1 boxplots (**c**) with the median, minimum, maximum, 25 and 75% interquartile range of each group, i.e., normal and cancer samples. Clathrin was overexpressed, significantly (****p < 0.0001) in cancer tissue samples, and there was a simultaneous and significant (****p < 0.0001) decrease of the expression of caveolin-1 in cancer tissue. Statistical significance for the difference in expression between normal and cancer samples was measured using Mann-Whitney *U* test

Tissue arrays were probed, simultaneously, with anti-clathrin and anti-caveolin-1 antibodies; both these proteins were found to be expressed in prostate tissue (Fig. 1). There was a visible increase in the expression of clathrin in cancerous prostate compared to normal tissue. Representative tissue cores for normal and cancer prostate tissue with clathrin (red) and caveolin-1 (green) show an increase in the expression of the former and corresponding decrease of expression of the latter in cancer tissue (Fig. 1). We used an ImageJ (Fiji)-based image quantitation method, developed in our laboratory (Arya et al. 2015), to quantify the expression signal for clathrin and caveolin-1. What was seen by eye (Fig. 1) was confirmed upon quantitation (Fig. 2a, b and c).

When a normal vs. cancer comparison was made for the expression of two proteins, there was a significant (*p* < 0.0001, Mann-Whitney) 2.5-fold increase in the expression of clathrin in prostate cancer tissue compared to corresponding controls (Fig. 2a); also, there was a concurrent and significant ~ 2.2 fold (*p* < 0.0001) decrease in the expression of caveolin-1 in prostate cancer tissue compared to normal tissue (Fig. 2b). The interquartile range of the expression of clathrin and caveolin-1 is shown in Fig. 2c. These results show, for the first time, that as there is an increase of clathrin protein expression in prostate cancer tissue, there is a concurrent, significant and opposite decrease in the expression of caveolin-1.

### Clathrin and caveolin-1 expression in low-, intermediate- and high-grade prostate cancer

**Fig. 3:**
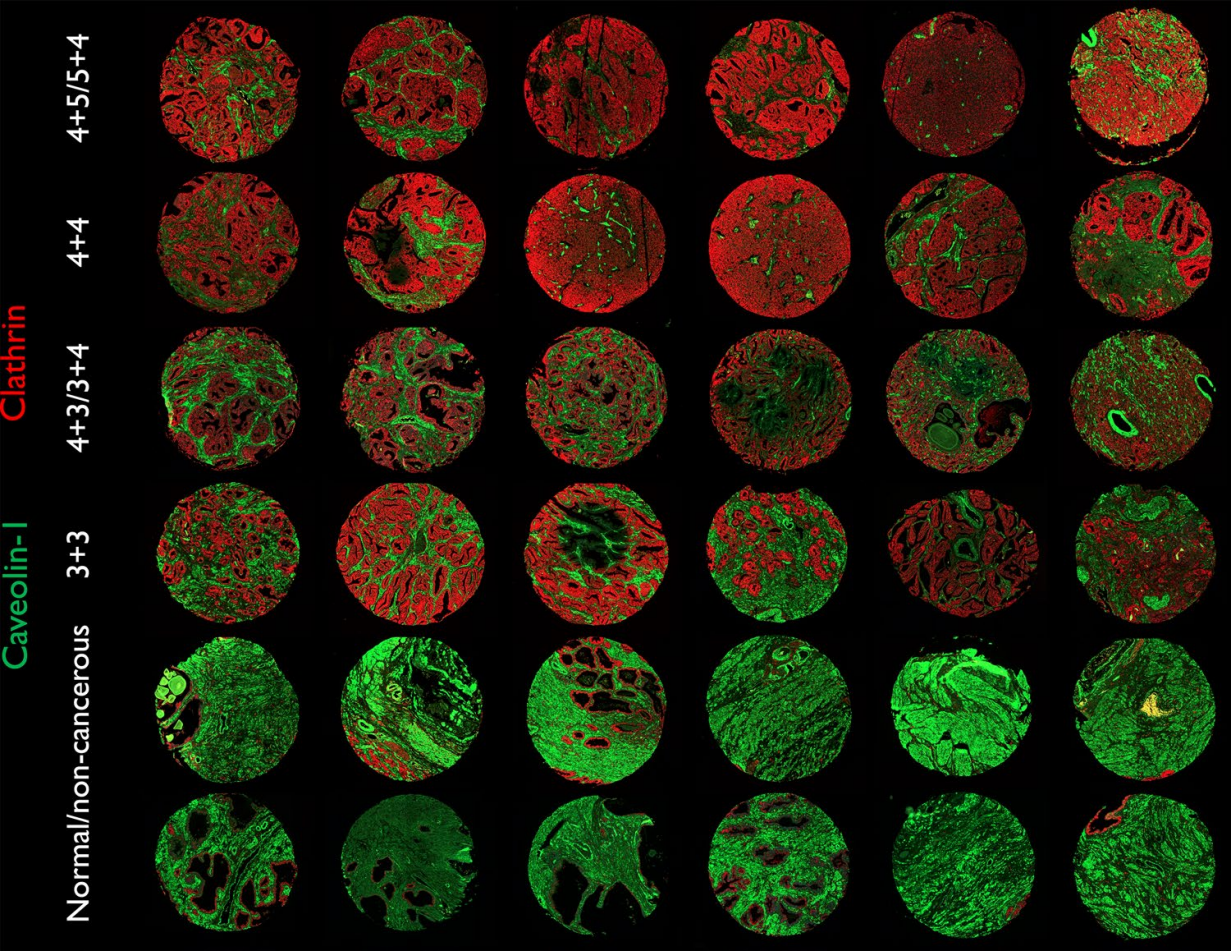
An example of prostate tissue used in this study from normal and cancer tissue with different Gleason scores (3 + 3, 3 + 4/4 + 3, 4 + 4, 4 + 5/5 + 4) probed with anti-clathrin (red) and anti-caveolin-1 (green) antibodies. All cores were imaged under Opal-520 (green, caveolin-1) and Opal-650 (red, clathrin) channels at 20 × magnification using an AxioScan Z1 (Zeiss). All settings were kept the same for a comparative analysis. Tissue core diameter: 1 mm

**Fig. 4:**
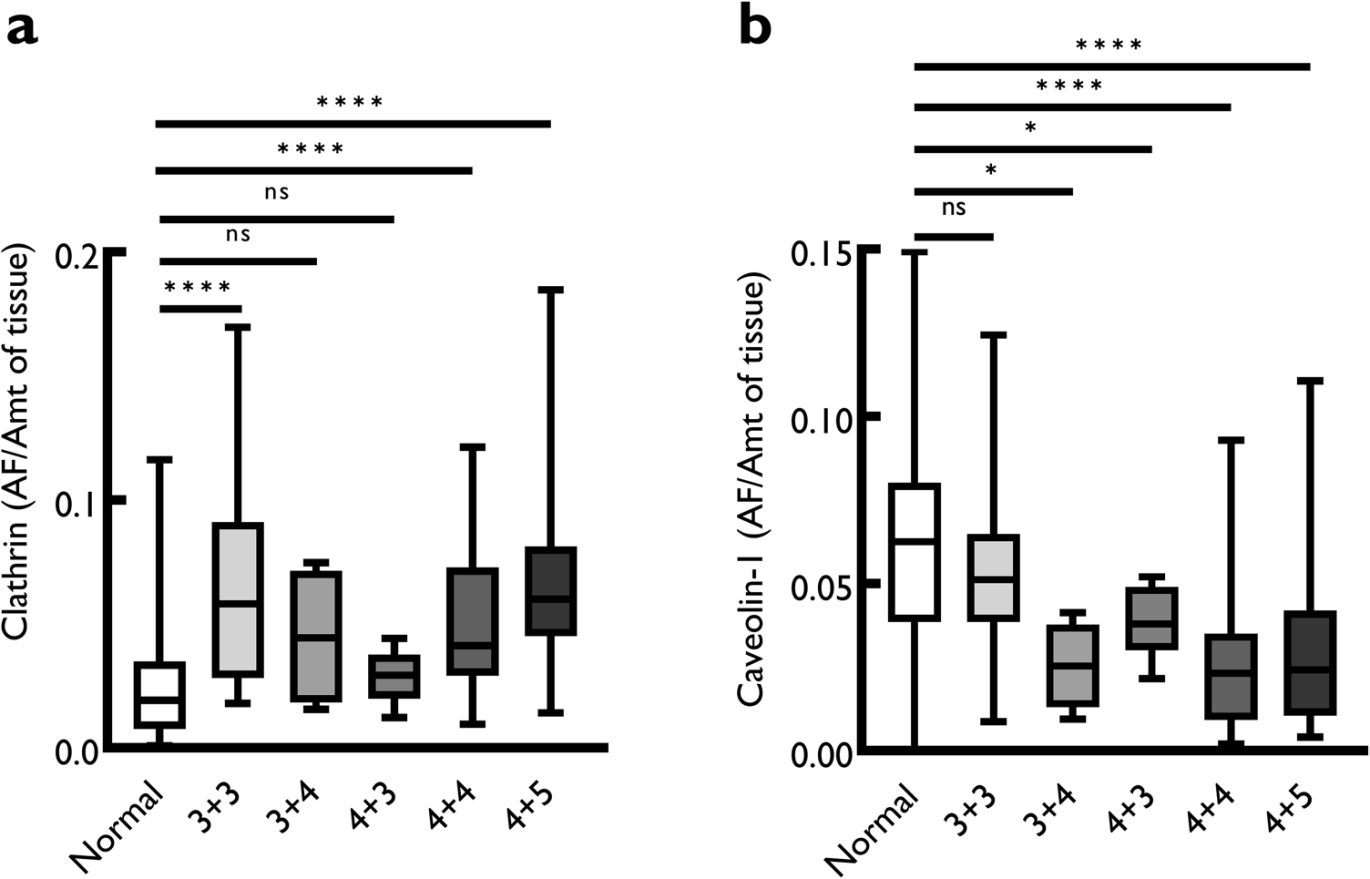
Boxplots for the quantitative expression of signal intensity of clathrin (**a**) and caveolin-1 (**b**) in cancer with different Gleason score compared to normal subsequent to analysis using ImageJ. Boxplots show the median, minimum, maximum, 25 and 75% interquartile range of each group. Results are expressed as signal expressed as area fraction (AF) divided by the amount (Amt) of tissue and fitted to a probit regression. Area fraction (AF) was divided by the total amount (Amt) of tissue in a core to standardize the data for the amount of tissue that was used in each core on the arrays. There is a trend towards increase in clathrin expression with progressing Gleason score, whereas caveolin-1 expression decreases progressively with increasing Gleason score. Significance was measured using Mann-Whitney *U* test using MedCalc software (ns = not significant; *p ≤ 0.05; **p ≤ 0.01; *** p ≤ 0.001; **** p ≤ 0.0001)

Prostate tissue used in the analysis above (Supplementary Fig. 1 and Supplementary Fig. 2, Figs. 1 and 2) contained cancer samples of different Gleason grades (see also Supplementary Table 1) that characterize the diagnosis of prostate cancer. Figure 3 shows a selection of prostate tissue used in this study probed with anti-clathrin (red) and anti-caveolin-1 (green) antibodies. There is a gradual increase in the expression of clathrin from normal, low-grade (3 + 3), intermediate-grade (3 + 4/4 + 3) to high-grade (4 + 4 and 4 + 5/5 + 4) prostate cancer. Although clathrin appears to be expressed *mostly* in the epithelium, caveolin-1 can be seen in both the stroma and acinar epithelium. Images including the ones shown here were used to quantify the expression of clathrin and caveolin-1 in different Gleason grade samples (Fig. 4).

Clathrin was significantly overexpressed in different Gleason grades of prostate cancer except 3 + 4/4 + 3 (Fig. 4a). For caveolin-1, although there was no significant difference between normal and Gleason 3 + 3 group (*p* > 0.05), there was a significant decrease in caveolin-1 expression with increasing Gleason score: Gleason 3 + 4 (*p* = 0.02), 4 + 3 (*p* = 0.03); Gleason 4 + 4 (*p* < 0.0001) and Gleason 4 + 5/5 + 4 (*p* < 0.0001) (Fig. 4b).

Segregating samples according to different Gleason scores and comparing these against normal tissue show that (1) clathrin expression shows a decrease in score 3 + 4 and 4 + 3 but the expression reverts back up to the levels of 3 + 3 Gleason grade in high Gleason grade cancer (Fig. 4a) and (2) there is a gradual and persistent decrease in the expression of caveolin-1 with increasing Gleason score (Fig. 4b). This indicates that prostate cancer progression from low Gleason grade may require an increase in clathrin and by extension CME, which may again be necessary for pre-metastatic cancer. Noting that there is a general decrease in caveolin-1 expression compared to normal prostate tissue (Fig. 2b), these results suggest that caveolin-1 plays a key role in the early carcinogenesis process, perhaps as a suppressor of tumorigenesis. These results show that in prostate cancer, the combination of a gradual decrease in caveolin-1 and increase in clathrin expression cancer may be critical for cancer progression.

### Colocalization analysis of clathrin and caveolin-1 in normal and cancerous prostate tissue

**Fig. 5:**
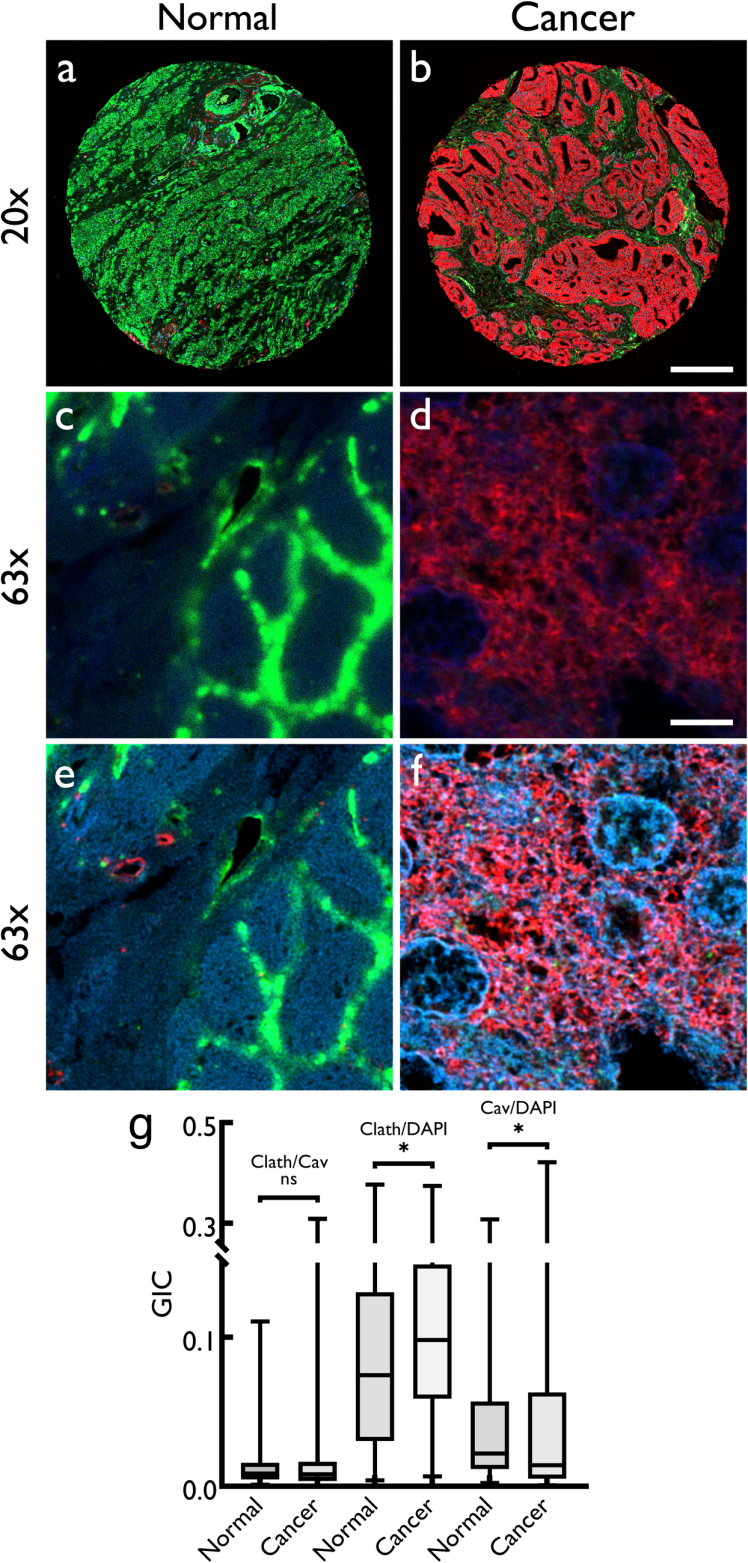
Representative confocal micrographs of normal (**a**) and cancer (**b**) human prostate tissue cores (at 20 × magnification) stained for clathrin (Opal-650, 561/595 nm, red), caveolin-1 (Opal-520, 488/517 nm, green) and cell nuclei (DAPI, 405/429 nm, blue). The cores (**a** and **b**) were scanned using AxioScan Z1 fluorescence slide scanner (Zeiss). The composite images of three channels clathrin (red), caveolin-1 (green) and DAPI (blue) are shown. **c** and **d** Maximum projection of Z-stacks of area from the same tissue cores imaged at 63 × magnification using an SP8 confocal microscope (Leica) with 6 × digital zoom with Z-section step size set to 0.17 μm, yielding approximately 26–30 Z-sections. The high magnification images were deconvolved (**e** and **f**) using Huygens Professional software (SVI) and analyzed for colocalization (global intersection coefficient) between two channels. The quantitative data for the global intersection coefficient (GIC) are shown as boxplot (**g**) as a comparison between normal and cancer for colocalization between clathrin/caveolin-1, clathrin/nuclei and caveolin-1/nuclei with median, minimum, maximum, 25 and 75% interquartile range of each group. Significance of difference between groups was measured using Mann-Whitney *U* test using MedCalc software (ns = not significant; *p ≤ 0.05). Scale bars: **b** 200 μm; **d** 6 μm

We have previously shown that in addition to protein expression, colocalization of different proteins may serve as an additional differential parameter and biomarker between non-cancerous and cancerous tissue samples (Arthurs et al. 2020; Symes et al. 2013). There are no reports regarding colocalization of clathrin and caveolin-1 in human prostate tissue or whether these are closely or distantly localized. To measure the colocalization of these two proteins, we imaged the tissue cores at a high magnification (× 63) using confocal microscopy. Figure 5 shows tissue cores at low magnification (20×, Fig. 5a and b) and areas of the same cores (see methods) imaged at 63 × magnification (Fig. 5c and d, maximum projection of confocal Z-stack). Deconvolution analysis using Huygens (SVI) deconvolution software was performed on high-magnification, high-resolution confocal images for the calculation of the GIC that estimates the proximity of pixels for two channels (Fig. 5e and f). We also used DAPI (a nuclear marker) to stain the nuclei and estimate the colocalization of clathrin and caveolin-1 with nuclei (Fig. 5g). There was no significant difference in the colocalization of clathrin and caveolin-1 in normal compared to cancer prostate tissue samples (Fig. 5g). Furthermore, there was no difference between the GIC for clathrin and caveolin-1 across tumors with different Gleason scores (Supplementary Fig. 3a). However, our results show a significant (*p* = 0.02) increase in the colocalization between clathrin and DAPI in cancerous compared to normal tissue (Fig. 5g). The GIC for clathrin and DAPI increased in Gleason score 3 + 3 and 4 + 4 cancer tissue compared to normal (Supplementary Fig. 3b). This indicates that clathrin may influence transcriptional processes during carcinogenesis. Conversely, decreased caveolin-1/DAPI GIC values were found in cancer compared to normal tissue (*p* = 0.03), indicating a loss of caveolin expression in the nucleus in cancer. This further suggests a putative tumor suppressor role of caveolin-1. GICs between different Gleason scores are also shown in Supplementary Fig. 3c; however, it is only indicative and would require further investigations to show a role in transcription in prostate cancer for either clathrin or caveolin-1.

### Clathrin, caveolin-1 and EGFR in prostate cancer

**Fig. 6:**
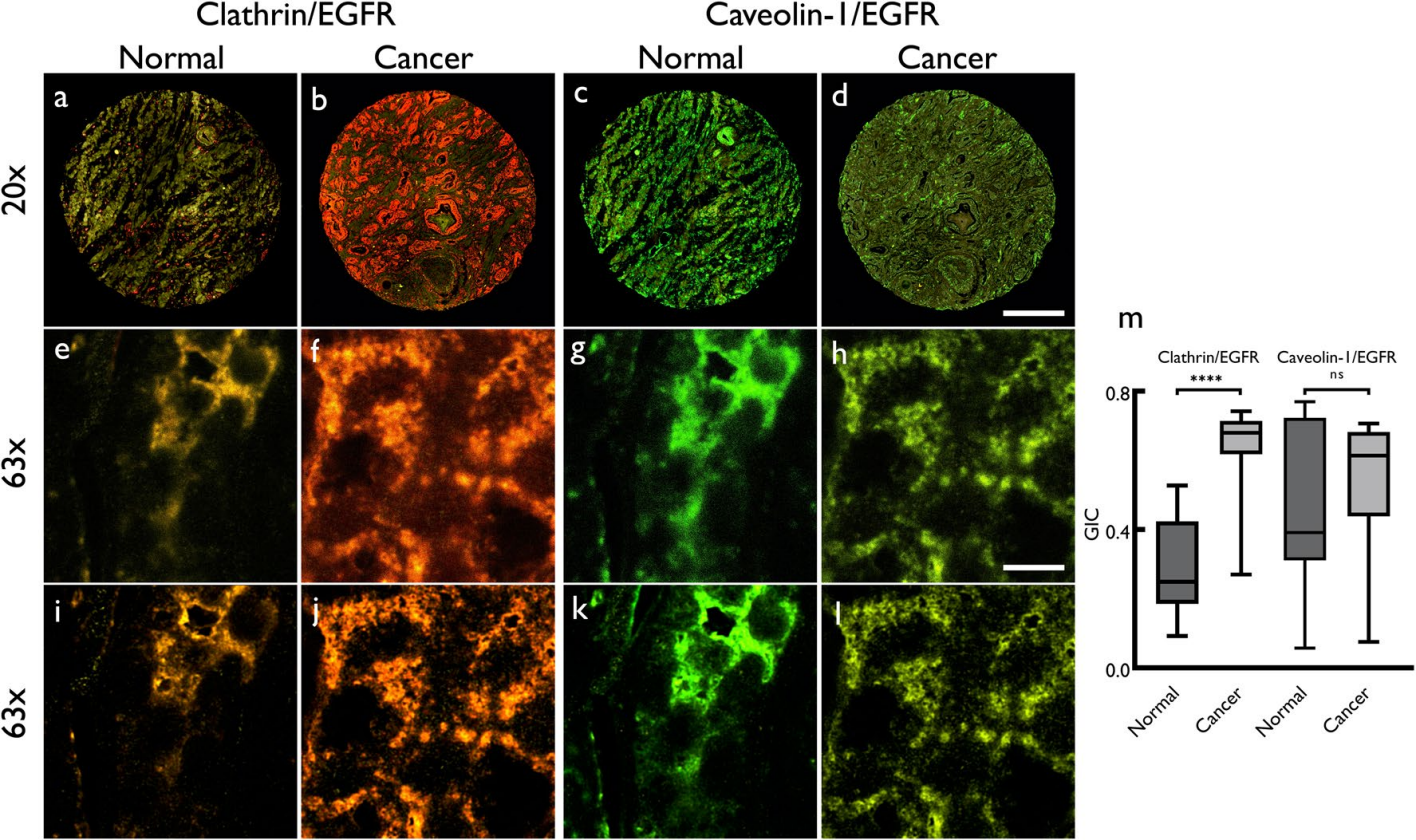
Representative confocal micrographs of normal (**a** and **c**) and cancer (**b** and **d**) human prostate tissue cores (at 20× magnification) stained for clathrin (561/595 nm, red), caveolin-1 (488/517 nm, green) and EGFR (577/603 nm, yellow) proteins and scanned using AxioScan Z1 fluorescence slide scanner (Zeiss); composite images of two channels: clathrin (red) and EGFR (yellow) and caveolin-1 (green) and EGFR (yellow) proteins are shown. **e**–**h** Area from the same tissue cores imaged at 63 × magnification (**e**–**h**) using an SP8 confocal microscope (Leica) with 6 × digital zoom with Z-section step size set to 0.17 μm, yielding approximately 26–30 z-sections. The high magnification images were deconvolved (**i–l**) using Huygens Professional software (SVI) and analyzed for colocalization (global intersection coefficient between two channels). The quantitative data for the global intersection coefficient (GIC) are shown as boxplot (**m**) as a comparison between normal and cancer for clathrin/EGFR and caveolin-1/EGFR with median, minimum, maximum, 25 and 75% interquartile range of each group. Significance of difference between groups was measured using Mann-Whitney *U* test using MedCalc software (ns = not significant; ****p ≤ 0.0001). Scale bars: **d** 300 μm; **h** 8 μm

To test the hypothesis that an important mechanism in prostate carcinogenesis is the receptor recycling for key cell proliferative pathways such as EGFR (Sigismund et al. 2008), we conducted simultaneous, multi-labeled staining of clathrin, caveolin-1 and EGFR. Figure 6 shows a normal and a cancer tissue core with combined clathrin/EGFR (Fig. 6a and b) and caveolin-1/EGFR channels (Fig. 6c and d). The expanded, high-magnification images from an area of the cores are shown in Fig. 6e–h. Deconvolved images, subsequent to high-resolution confocal microscopy, were used to measure GIC in prostate normal and cancer tissue (Fig. 6i–l). Quantitation of GIC between normal and cancer tissue in two protein pairs (clathrin/EGFR, caveolin-1/EGFR) is also shown (Fig. 6m); single-channel images are shown in Supplementary Fig. 4. These results indicate a significant increase of the colocalization between clathrin and EGFR in cancerous tissue compared to normal (*p* < 0.0001) (Fig. 6m), indicating that clathrin may play an important role in facilitating EGFR recycle via CME process.

### Clathrin, EGFR and AP2 in prostate cancer

**Fig. 7:**
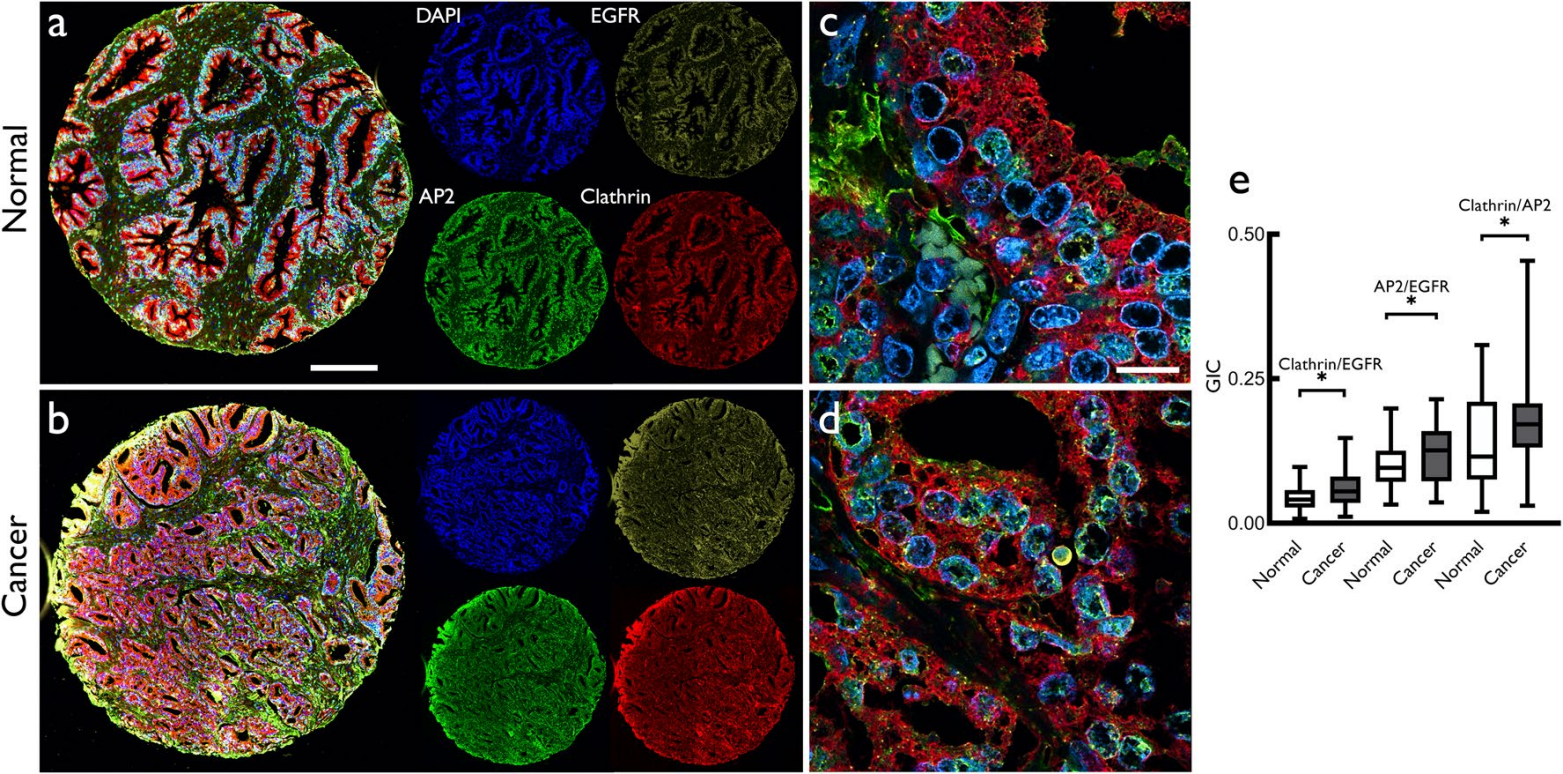
Representative confocal micrographs of **a** normal **b** cancer human prostate tissue cores showing images (at 20 × magnification using AxioScan Z1 scanner) for DAPI (353/465 nm, blue), EGFR (577/603 nm, yellow), AP2 (488/517 nm, green) and clathrin (561/595 nm, red) proteins. The corresponding, deconvolved high magnification (63×) confocal images **c** and **d** show epithelial tissue area of the 20× magnified tissue cores. Confocal images (from a Leica SP8 microscope) were deconvolved using Huygens Professional software (SVI) and analyzed for colocalization (global intersection coefficient between two given channels). The quantitative data for the global intersection coefficient (GIC) are shown as boxplot (**e**) for normal and cancer for clathrin/EGFR and AP2/EGFR and clathrin/AP2. Significance of difference between groups was measured using Mann-Whitney *U* test using MedCalc software (*p ≤ 0.05). Scale bar: **a** 200 μm; **c** 14 μm

Adaptor protein complex cargo into clathrin-coated pits. Proteins such as the AP2 complex, AP180, epsin and stoning are key clathrin adaptor proteins. Of these, AP2 is an abundant adaptor protein, is used as a clathrin pit marker (Santini and Keen 1996) and is also a regulator of CME (Kadlecova et al. 2017). We therefore used AP2 in conjunction with clathrin and EGFR on human prostate tissue arrays. Figure 7 shows the expression of clathrin, EGFR and AP2 in representative normal (Fig. 7a) and cancer (Fig. 7b) tissue cores assessed using fluorescence imaging. We also performed confocal imaging of the tissue cores stained for clathrin, EGFR and AP2 and deconvolved these for colocalization analysis (Fig. 7c, d and e).

The colocalization coefficients (GIC) of clathrin and EGFR, clathrin and AP2 and EGFR and AP2 all showed a significant increase in cancer compared to normal (*p* = 0.0125, *p* = 0.0125, *p* = 0.0128, respectively). These results support the notion that there may be an increase in endocytotic clathrin complexes in addition to a general increase in clathrin expression in prostate cancer. These data further support the proposition that there may be an increase in EGFR recycling via CME in prostate cancer as a mechanism that amplifies carcinogenesis.

## Discussion

In this investigation we show, for the first time to our knowledge, an opposite expression of the proteins involved in two key endocytotic pathways, namely CME and CavME. We demonstrate that there is an increase in the expression of clathrin with increasing Gleason score in prostate cancer and a concurrent decrease in caveolin-1 expression. The expression of clathrin is almost exclusively found in epithelial cells in both normal and cancer samples whereas caveolin-1, in addition to being expressed largely in stromal tissue, also shows expression in epithelial cells (Figs. 1, 3, 5, 6 and 7) in normal tissue. The overall increase in the expression of clathrin in prostate cancer (Fig. 2) could be explained by the increase in the number of epithelial acini as the cancer progresses. A simple correlative conclusion of these results appears to be that caveolin-1 expression and by extension CavME may act as a brake on tumorigenesis; a decrease in caveolin-1 expression may accelerate tumorigenesis, which is further enhanced by overexpression of clathrin with its known function in receptor recycling for key carcinogenic pathways (Imbastari et al. 2021).

Indeed, an interesting aspect of CME and clathrin independent endocytosis is the targeted internalization of key receptor signaling involved in cell proliferation (Brunt and Scholpp 2018; Perez Verdaguer et al. 2021; Wee and Wang 2017). In the context of receptor-mediated cell signaling, endocytosis may (1) attenuate an extracellular signal or (2) facilitate signal reactivation via receptor recycling back to the cell surface. We discuss some of the ramifications of our results in prostate cancer and provide a framework for the putative mechanisms that may also be relevant to other cancers.

### CME activates proliferative signaling pathways

Cancer metastasis is a complex, incremental process, partly a result of interaction between tumor cells and their microenvironment. Endocytosis is a major regulator of receptor availability for these signaling pathways (Khan and Steeg 2021; Mellman and Yarden 2013). As a result, there is emerging interest in endocytosis as a key process associated with carcinogenesis, reduced tumor suppression and metastasis in different cancer types (Mellman and Yarden 2013). We discuss these notions within the framework of two key carcinogenic EGFR signaling. Various elements of Wnt signaling are known to be mutated in epithelial cancers, including those of the prostate. Furthermore, EGFR signaling has been the subject of many investigations regarding the role of RMEs (Khan and Steeg 2021).

Proliferative signaling processes, such as the EGFR pathway, also require endocytosis for signal activation that result in increased transcriptional activity of proto-oncogenes. It has been suggested that nearly 20% of all prostate cancers may have activated EGFR signaling (Hashmi et al. 2019). An interesting mechanism of CME, proposed by Sigismund and colleagues (Sigismund et al. 2008), is that EGFR is internalized by CME, but rather than being degraded, these receptors are recycled to the cell membrane. Our results suggest that, in prostate cancer, increase in clathrin expression is associated with an increased colocalization with EGFR (Fig. 6), indicating EGFR recycling may allow cancer to progress through a constitutive proliferative signaling mechanism.

Other signaling pathways such as the Wnt signaling network also play an important role in carcinogenesis and cancer metastasis (Murillo-Garzon and Kypta 2017). Wnt proteins are upregulated in prostate and other cancers (Wang et al. 2010; Yang et al. 2006) and play a key role in cell proliferation. Wnt ligands (e.g., Wnt3A, 4, 5A, 7, 9B) act as paracrine signals, produced from source cells, and act on their target cells (Brunt and Scholpp 2018). It is known that Wnt signaling is under the regulation of CME (Blitzer and Nusse 2006). CME of Wnt/Fzd receptors is thought to enhance Wnt/β-catenin signal (Brunt and Scholpp 2018), although how endocytosis regulates or is regulated by Wnt signaling is not clear. However, the mechanism of CME regulation of Wnt signaling is different from that proposed for other signaling mechanisms such as EGFR—unlike the receptor recycling for EGFR activation by CME (see below), endocytosis of Wnt ligands occurs in the source cells (Brunt and Scholpp 2018; Sigismund et al. 2018). A further interesting aspect of endocytosis control of Wnt signaling is the suggestion that Wnt3A-LRP6 receptor complex is internalized by CavME. Caveolin-1 is also reduced in other cancers such as those of the breast, liver and ovary [reviewed in (Goetz et al. 2008)]. Considering the role of CME and CavME in EGFR and Wnt signaling, our data (Figs. 2 and 3) yield an interesting hypothesis: the decline in the expression of caveolin-1 may be acting as a tumor suppressor whereas clathrin overexpression in progressive grades of prostate cancer may suggest a tumor promotion role through its regulation of Wnt signaling. A similar role could also be construed for Erb2 signaling (Hynes and MacDonald 2009).

In summary, we present data that suggest a novel role of endocytotic pathways in human prostate cancer. Our results indicate that CME and CavME may act in concert in prostate carcinogenesis and progression. These results highlight the central role of endocytosis in prostate cancer and suggest the possibility of endocytosis pathways as a disease stratification biomarker and a target for therapy of prostate cancer.

## Supplementary Information

Below is the link to the electronic supplementary material.

Supplementary file1 (TIFF 49106 KB)

Supplementary file2 (DOCX 9423 KB)

## Data Availability

All data is available through supplementary material.
