# Supplementary figures and images for: Opposite changes in the expression of clathrin and caveolin-1 in normal and cancerous human prostate tissue: putative clathrin-mediated recycling of EGFR
Source: Histochem Cell Biol. 2023 Mar 4;159(6):489–500. doi: 10.1007/s00418-023-02183-8 (PMC10247851; doi:10.1007/s00418-023-02183-8)

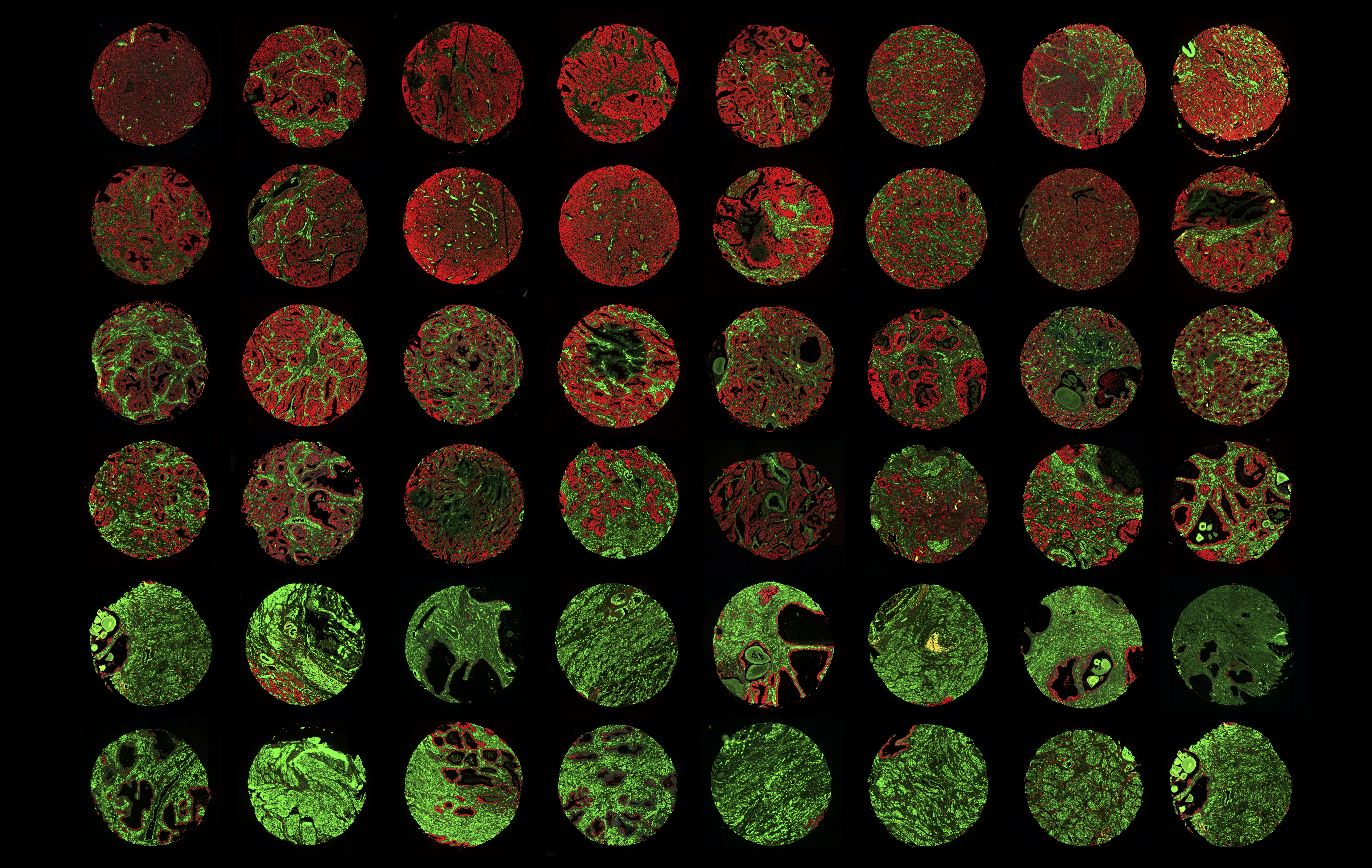

Supplement: Supplementary file 1 — Supplementary file1 (TIFF 49106 KB) [file 418_2023_2183_MOESM1_ESM.tiff]
