# Supplementary material for: Opposite changes in the expression of clathrin and caveolin-1 in normal and cancerous human prostate tissue: putative clathrin-mediated recycling of EGFR
Source: Histochem Cell Biol. 2023 Mar 4;159(6):489–500. doi: 10.1007/s00418-023-02183-8 (PMC10247851; doi:10.1007/s00418-023-02183-8)
Supplement: Supplementary file 2 — Supplementary file2 (DOCX 9423 KB) [file 418_2023_2183_MOESM2_ESM.docx]

**Supplementary Information**

Supplementary Table 1. Patient information for this study includes patient ID, serum prostate specific antigen (PSA) values at diagnosis, Gleason grades, grade group, pT stage, Tstage and age of diagnosis. Number of normal and cancer tissue array (TA) cores generated from each patient is also shown.

| **Patient ID (*N*=27)** | **Serum PSA at diagnosis** | **Gleason Grades** | **Grade Group** | **pT Stage** | **TStage** | **Age at diagnosis** | **Number of normal TA core generated (*n*=68)** | **Number of cancer TA core generated (*n*=93)** | **Gleason grades of cancer cores** |
| --- | --- | --- | --- | --- | --- | --- | --- | --- | --- |
| **H0705054** | 3.95 | 4+5 | 5 | 3 | 3 | 61 | 1 | 4 | 4+4 |
| **H0706129** | 3.30 | 4+5 | 5 | 3 | 3 | 70 | 2 | 3 | 4+4 |
| **H0707037** | 15.00 | 4+4 | 4 | 2 | 2 | 60 | 1 | 3 | 1x 3+4; 2x 4+3 |
| **H0708039** | 4.81 | 5+4 | 5 | 2 | 2 | 67 | 2 | 3 | 2x 4+5; 1x 5+4 |
| **H0708591** | 10.02 | 5+4 | 5 | 4 | 4 | 62 | 1 | 5 | 4+4 |
| **H0709209** | 8.32 | 4+4 | 4 | 2 | 2 | 65 | 2 | 3 | 1x 3+4; 2x 4+4 |
| **H0800120** | 5.00 | 4+4 | 4 | 2 | 2 | 49 | 2 | 3 | 4+3 |
| **H0800366** | 6.00 | 4+4 | 4 | 2 | 2 | 65 | 1 | 3 | 4+4 |
| **H0800717** | 9.39 | 4+4 | 4 | 3 | 3 | 47 | 2 | 3 | 1x 4+3; 2x 4+4 |
| **H0801278** | 5.20 | 4+4 | 4 | 2 | 2 | 60 | 1 | 4 | 1x 4+3; 1x 4+4; 2x 4+5 |
| **H0801755** | 12.20 | 4+5 | 5 | 1 | 1 | 64 | 2 | 5 | 1x 4+4; 2x 4+5 |
| **H0803643** | 10.30 | 4+5 | 5 | 2 | 2 | 62 | 2 | 3 | 1x 3+4; 2x 4+4 |
| **H0805622** | 6.11 | 4+5 | 5 | 2 | 2 | 58 | 2 | 3 | 4+4 |
| **H0806495** | 7.29 | 4+4 | 4 | 2 | 2 | 67 | 2 | 3 | 4+4 |
| **H0901804** | 9.80 | 3+3 | 1 | 2 | 2 | 65 | 3 | 3 | 3+3 |
| **H0912369** | 12.00 | 3+3 | 1 | 2 | 2 | 54 | 3 | 3 | 3+3 |
| **H1103610** | 7.07 | 3+3 | 1 | 2 | 2 | 50 | 3 | 3 | 3+3 |
| **H1104892** | 2.46 | 3+3 | 1 | 2 | 2 | 51 | 3 | 3 | 3+3 |
| **H1109027** | 4.41 | 3+3 | 1 | 2 | 2 | 63 | 3 | 3 | 3+3 |
| **H1307652** | 13.10 | 4+4 | 4 | 3 | 3 | 61 | 3 | 3 | 4+4 |
| **H1307856** | 8.80 | 4+4 | 4 | 2 | 2 | 49 | 3 | 3 | 4+4 |
| **H1308297** | 6.97 | 4+4 | 4 | 3 | 3 | 54 | 3 | 3 | 4+4 |
| **H1313345** | 9.95 | 4+4 | 4 | 3 | 3 | 68 | 3 | 3 | 4+4 |
| **H1005541** | 7.25 | 4+4 | 4 | 3 | 3 | 48 | 3 | 3 | 4+4 |
| **H0810764** | 18.80 | 4+5 | 5 | 2 | 2 | 60 | 3 | 3 | 4+5 |
| **H0909375** | 7.14 | 4+5 | 5 | 3 | 3 | 55 | 3 | 3 | 4+5 |
| **H1313961** | 10.80 | 4+5 | 5 | 3 | 3 | 56 | 3 | 3 | 4+5 |
| **H1400020** | 10.00 | 4+5 | 5 | 3 | 3 | 59 | 3 | 3 | 4+5 |
| **H1400073** | 13.00 | 4+5 | 5 | 3 | 3 | 69 | 3 | 3 | 4+5 |


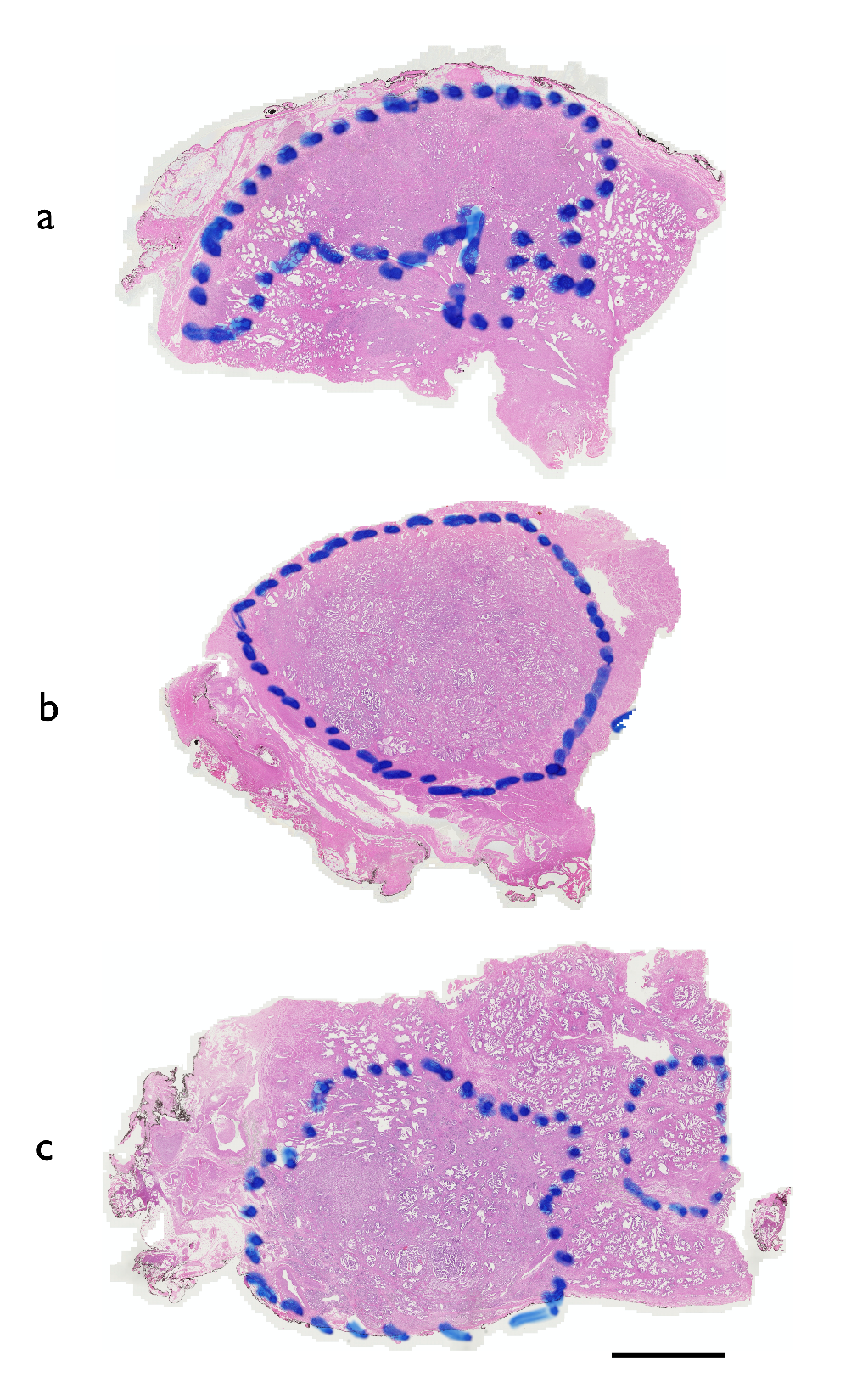


Supplementary Fig 1. Three representative examples (a, b and c) of the whole-face human prostate tissue sample sections that stained using Hematoxylin and Eosin. Areas circled in blue were identified by an expert pathologist (RH) as cancerous; areas outside blue mark are normal (cancer adjacent, non-cancerous) tissue. All images were obtained using AxioScan Z1 (Zeiss) at 20x magnification. Scale bar: 5 mm.

**a**

**
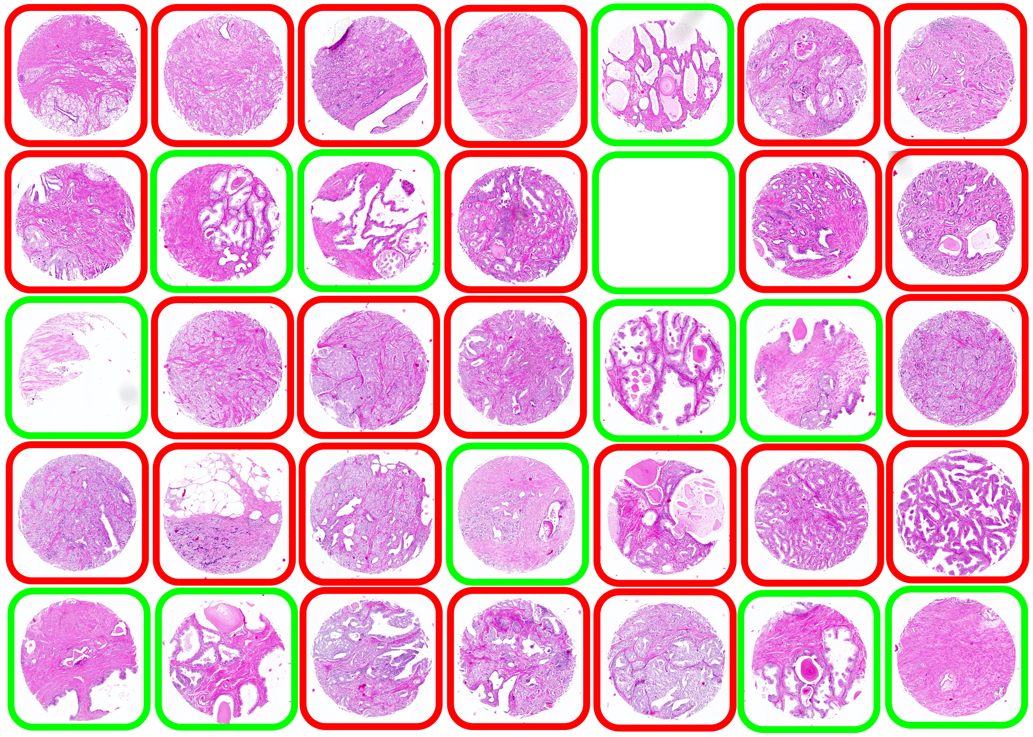
**

**b**

**
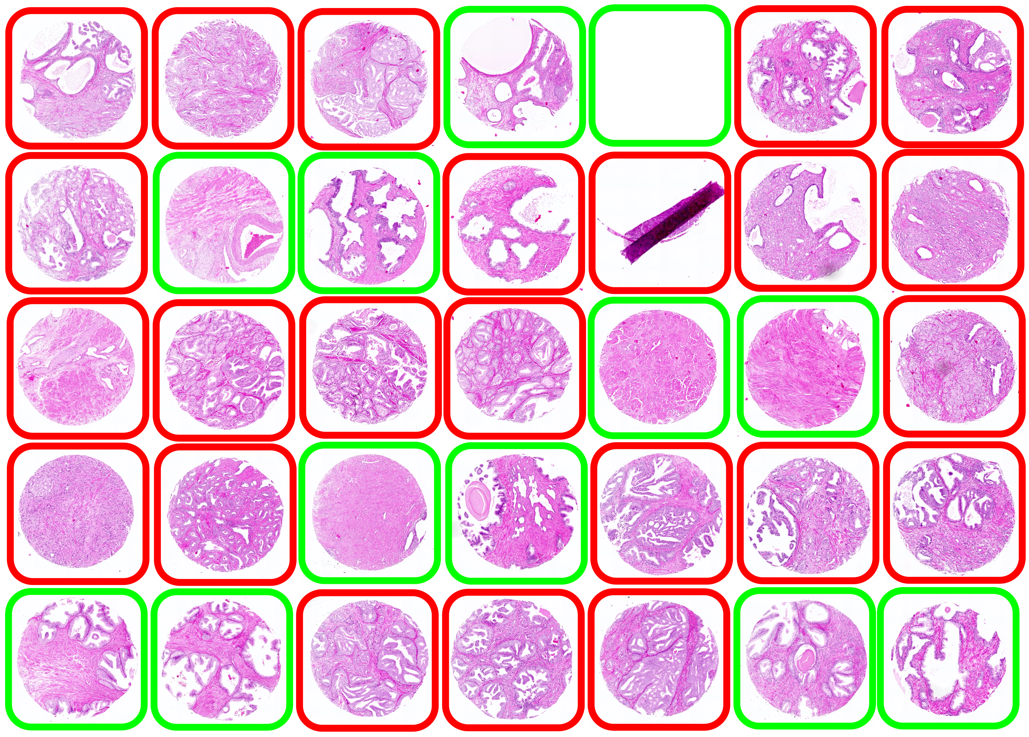
**

**c**


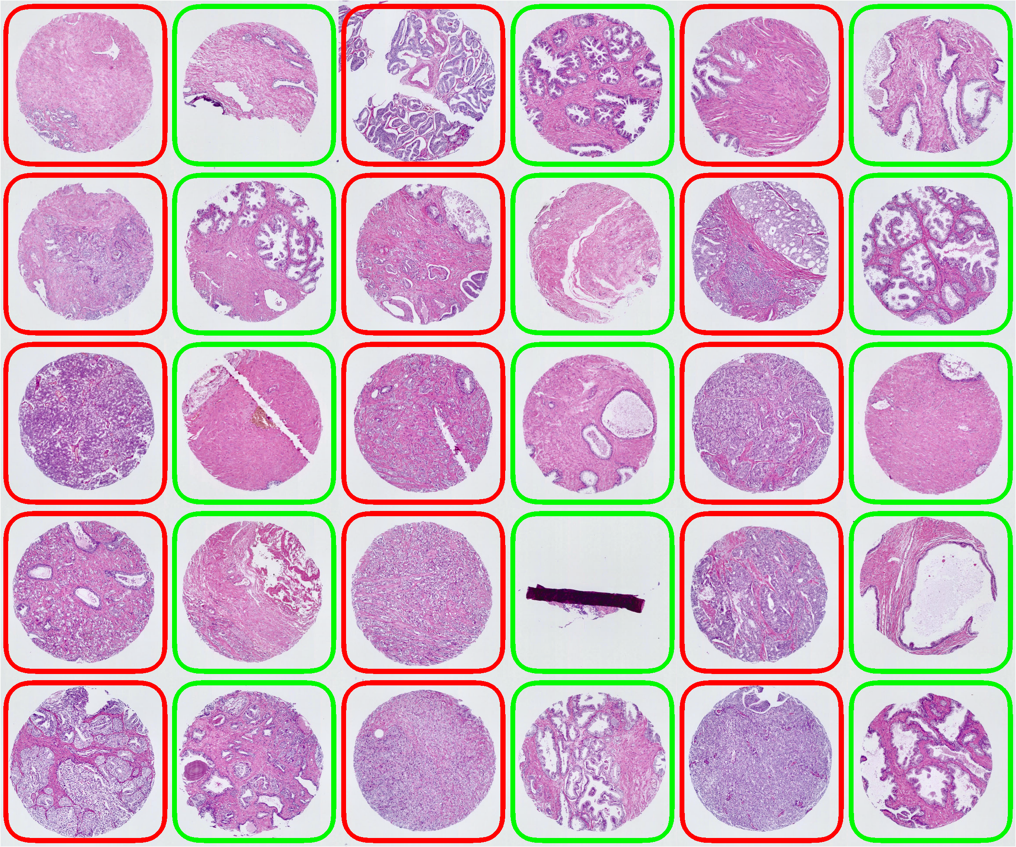


**d**


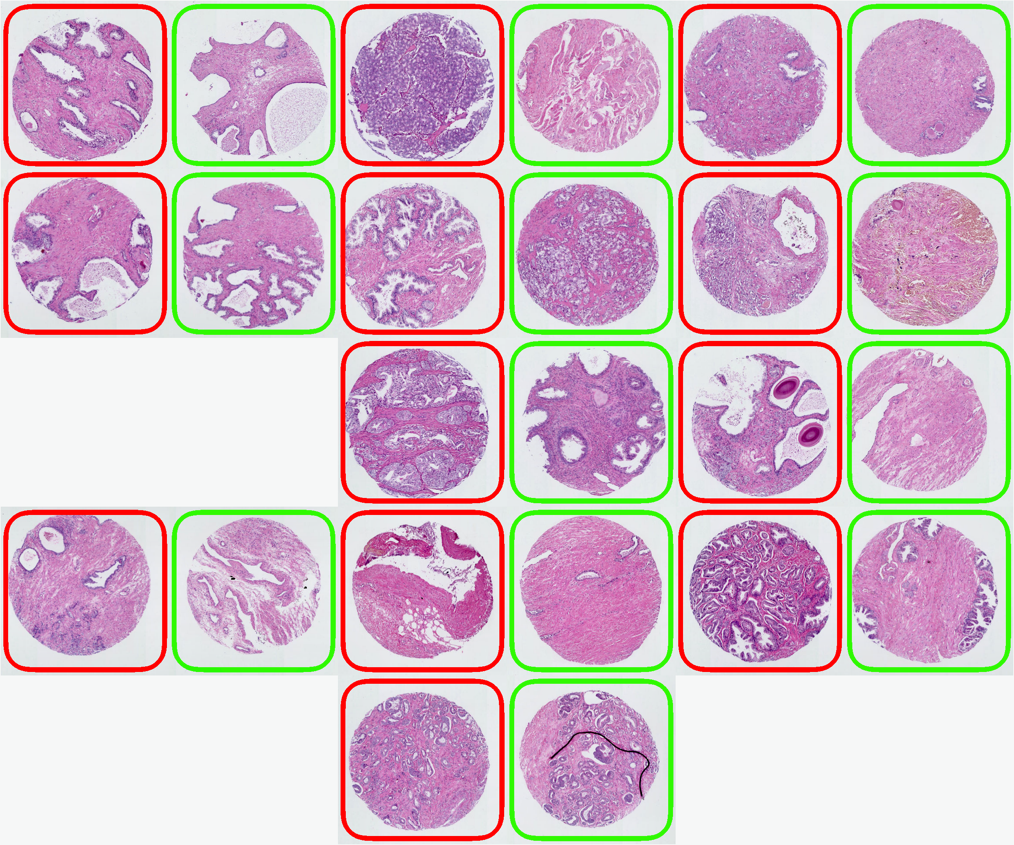


**e**


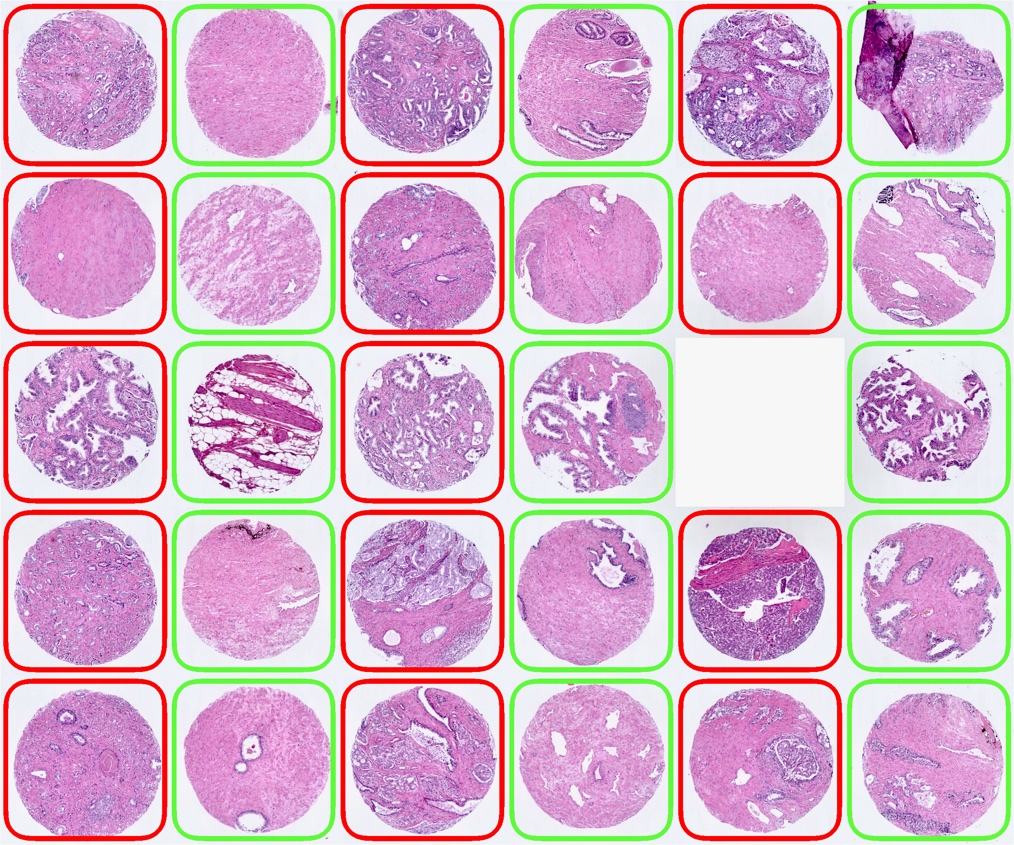


Supplementary Fig 2. Hematoxylin & Eosin stained human prostate tissue samples arrayed on the tissue arrays used in this study (a-e). Cancerous cores are framed in red, normal (cancer-adjacent, non-cancerous) cores in green. The tissue arrays were scanned using AxioScan Z1 (Zeiss) at 20x magnification. Diameter of each core: 1 mm.


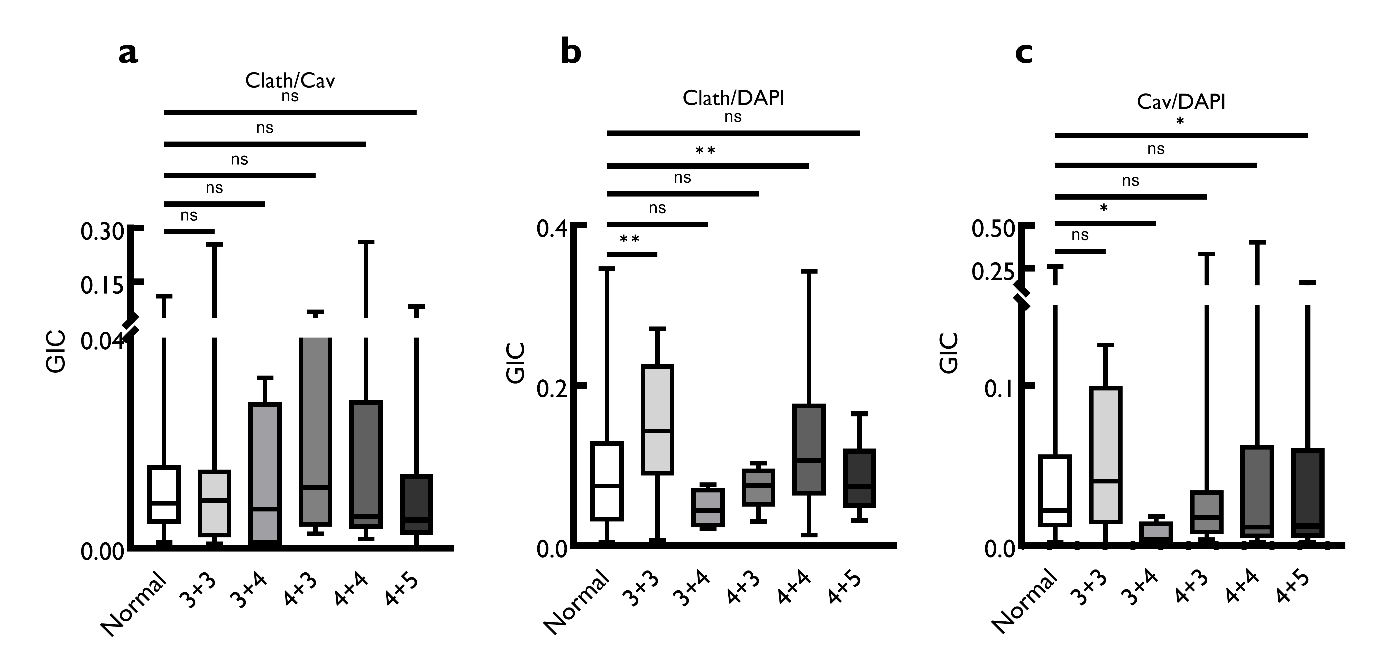


Supplementary Fig 3. Boxplots of Global Intersection Coefficient (GIC) between normal and cancer groups in different Gleason scores in different channel pairs: clathrin/caveolin-1 (a); clathrin/DAPI (b); and caveolin-1/DAPI (c). Statistical analysis was performed using Mann-Whitney U test using MedCalc software (ns = not significant; * = *p*≤0.05; ** = *p*≤0.01). This figure is supplement to Fig 5g in the main text.


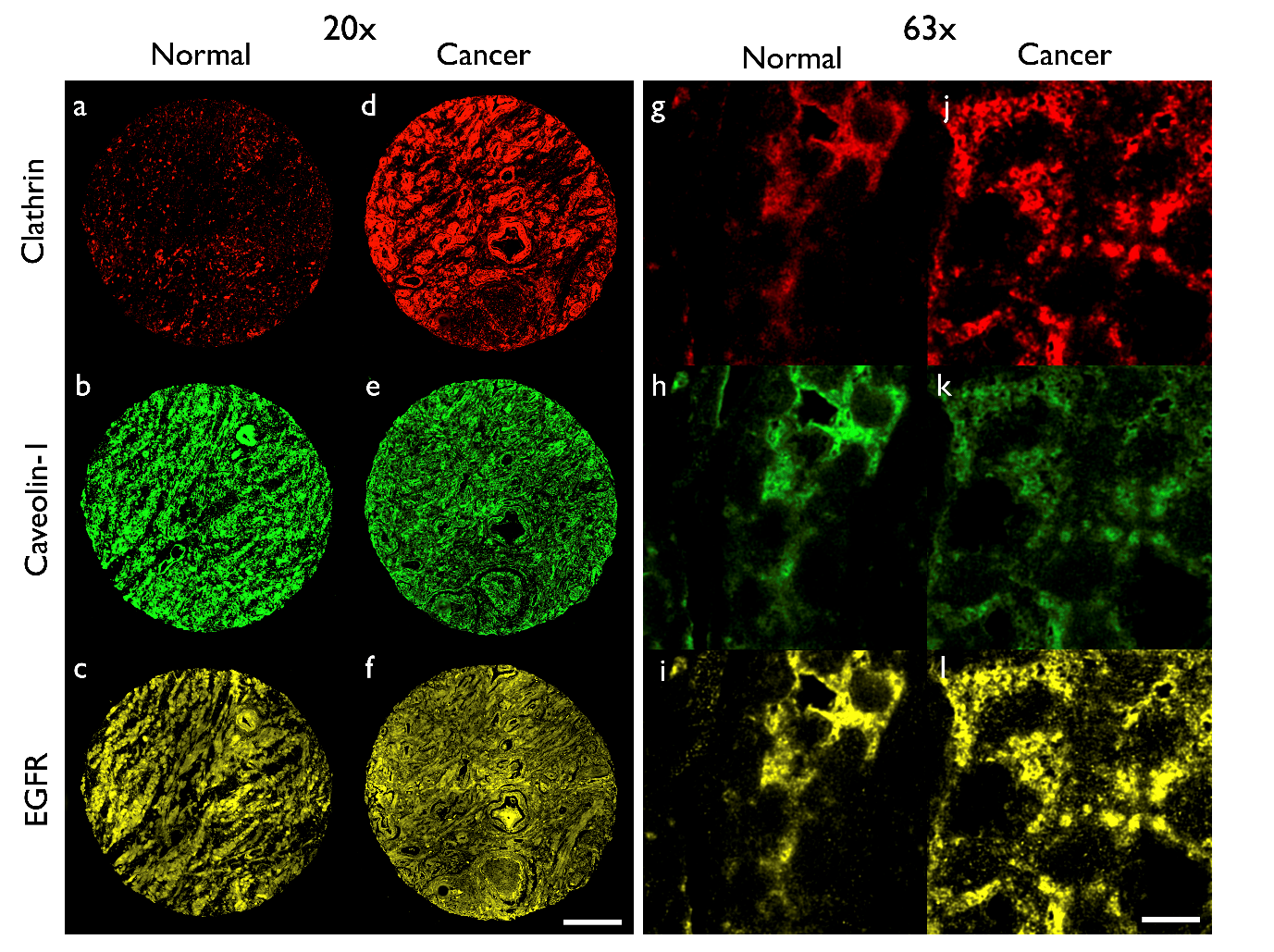


Supplementary Fig 4. Representative confocal micrographs of a normal (a-c) and a cancer (d-f) human prostate tissue cores (at 20x magnification) stained for clathrin (561/595nm, red), caveolin-1 (488/517nm, green) and EGFR (577/603nm, yellow) proteins and scanned using AxioScan Z1 fluorescence slide scanner (Zeiss); composite images of 2 channels clathrin (red) and EGFR and caveolin-1 (yellow) and EGFR proteins (green) are shown. g-l show an area from the same tissue cores deconvolved after imaging at 63x magnification using a SP8 confocal microscope (Leica) with 6x digital zoom with Z-section step size set to 0.17μm, yielding approximately 18–24 z-sections. The high magnification images were deconvolved using Huygens Deconvolution software (SVI) and analysed for colocalisation (Global intersection coefficient between two channels. Scale bars (f): 300 μm; (l) 8 μm.
